# Bile acids increase steroidogenesis in cholemic mice and induce cortisol secretion in adrenocortical H295R cells via S1PR2, ERK and SF‐1

**DOI:** 10.1111/liv.14052

**Published:** 2019-02-17

**Authors:** Lei Liu, Katrin Panzitt, Silvia Racedo, Martin Wagner, Wolfgang Platzer, Alex Zaufel, Verena Theiler‐Schwetz, Barbara Obermayer‐Pietsch, Helmut Müller, Gerald Höfler, Akos Heinemann, Gernot Zollner, Peter Fickert

**Affiliations:** ^1^ Research Unit for Experimental and Molecular Hepatology Division of Gastroenterology and Hepatology Department of Internal Medicine Medical University of Graz Graz Austria; ^2^ Research Unit for Translational Nuclear Receptor Research in Liver Metabolism Division of Gastroenterology and Hepatology Department of Internal Medicine Medical University of Graz Graz Austria; ^3^ Institute of Experimental and Clinical Pharmacology Medical University of Graz Graz Austria; ^4^ Division of Endocrinology and Diabetology Medical University of Graz Graz Austria; ^5^ Division of Transplant Surgery Medical University of Graz Graz Austria; ^6^ Institute of Pathology Medical University of Graz Graz Austria

**Keywords:** adrenal steroidogenesis, extracellular signal‐regulated kinase, sphingosine 1‐phosphate receptor 2, steroidogenic factor 1

## Abstract

**Background and Aims:**

Bile acids are now accepted as central signalling molecules for the regulation of glucose, amino acid and lipid metabolism. Adrenal gland cortex cells express the bile acid receptors farnesoid X receptor (FXR), the G protein‐coupled bile acid receptor (TGR5) and the sphingosine‐1‐phosphate receptor 2 (S1PR2). We aimed to determine the effects of cholestasis and more specifically of bile acids on cortisol production.

**Methods:**

FXR and TGR5 knockout mice and controls were subjected to common bile duct ligation (CBDL) or chenodeoxycholic acid (CDCA) feeding to model cholestasis. Human adrenocortical H295R cells were challenged with bile acids for mechanistic studies.

**Results:**

We found that CBDL and CDCA feeding increased the levels of corticosterone, the rodent equivalent to human cortisol and mRNA and protein levels of steroidogenesis‐related enzymes in adrenals independent of FXR and TGR5. Taurine‐conjugated CDCA (TCDCA) significantly stimulated cortisol secretion, phosphorylation of extracellular signal‐regulated kinase (ERK) and expression of steroidogenesis‐related genes in human adrenocortical H295R cells. FXR and TGR5 agonists failed to induce cortisol secretion in H295R cells. S1PR2 inhibition significantly abolished TCDCA‐induced cortisol secretion, lowered phosphorylation of ERK and abrogated enhanced transcription of steroidogenesis‐related genes in H295R cells. Likewise, siRNA S1PR2 treatment reduced the phosphorylation of ERK and cortisol secretion. Steroidogenic factor‐1 (SF‐1) transactivation activity was increased upon TCDCA treatment suggesting that bile acid signalling is linked to SF‐1. Treatment with SF‐1 inverse agonist AC45594 also reduced TCDCA‐induced steroidogenesis.

**Conclusions:**

Our findings indicate that supraphysiological bile acid levels as observed in cholestasis stimulate steroidogenesis via an S1PR2‐ERK‐SF‐1 signalling pathway.

**Abbreviations**
HPA: hypothalamic‐pituitary‐adrenal
CRH: corticotropin‐releasing hormone
ACTH: adrenocorticotropic hormone
CBDL: common bile duct ligation
CDCA: chenodeoxycholic acid
EGFR: epidermal growth factor receptor
FXR: farnesoid X receptor
CYP11B: cytochrome P450 11B, also referred as steroid 11β‐hydroxylase
CYP21: cytochrome P450 family 21, also referred to as steroid 21‐hydroxylase
HSD3B2: hydroxy‐delta‐5‐steroid dehydrogenase, 3 beta‐ and steroid delta‐isomerase 2
PBS: phosphate‐buffered saline
DMSO: dimethyl sulphoxide
GAPDH: glyceraldehyde 3‐phosphate dehydrogenase
ERK: extracellular signal‐regulated kinase
S1P: sphingosine 1‐phosphate
S1PR2: sphingosine 1‐phosphate receptor 2
SF‐1: steroidogenic factor 1
TCDCA: taurochenodeoxycholic acid
TGR5: transmembrane G protein‐coupled receptor, also known as GPBAR

**Key points**

- Experimental cholestasis and feeding bile acids in mice induce steroidogenesis independent of FXR and TGR5.
- Supraphysiological bile acid concentrations directly stimulate steroidogenesis in human adrenal H295R cells via an S1PR2‐ERK‐SF‐1‐dependent pathway.
- Our findings indicate that supraphysiological bile acid levels modulate adrenal gland function with potential important clinical implications.

## 1 INTRODUCTION

Bile acids are a heterogeneous family of complex molecules with steroidal structure and are actively secreted along with cholesterol and phospholipids into bile.^1^ In addition to their major function of facilitating digestion and absorption of nutrient lipids, bile acids are now also considered as signalling molecules and their receptors are *en vogue* targets for drug development. This is primarily related to the fact that bile acids are now known to regulate amino acid, lipid and glucose metabolism and consequently energy homoeostasis which are mainly mediated by bile acids’ characterized receptors: the farnesoid X receptor (FXR), the G protein‐coupled bile acid receptor (TGR5) and the sphingosine‐1‐phosphate receptor 2 (S1PR2).^2^, ^3^, ^4^ Significantly elevated serum bile acid levels (up to 30‐fold the upper limit of the normal range) are the hallmark of cholestatic liver diseases.^5^, ^6^ Bile acids activate a variety of defence mechanisms mediated by their receptors to reduce bile acid toxicity and to increase their elimination.^7^ Nevertheless, cholestatic disorders have a significant morbidity and mortality and liver transplantation is required when long‐standing cholestasis leads to end‐stage liver disease.

Glucocorticoids are essential for survival as they are key regulators of metabolism and stress response. The natural glucocorticoids (ie cortisol in humans, corticosterone in rodents) are synthesized in the *zona fasciculata* of the adrenal cortex. Illness and stress activate the hypothalamic‐pituitary‐adrenal (HPA) axis, causing secretion of corticotropin‐releasing hormone (CRH) from the hypothalamus leading to pituitary adrenocorticotropic hormone (ACTH) secretion. ACTH stimulates glucocorticoid synthesis in the adrenal cortex and glucocorticoids in turn regulate the activity of the HPA axis by acting on extrahypothalamic centres, the hypothalamus and the pituitary gland, thereby establishing a feedback loop.^8^

There is numerous clinical and experimental evidence tempting to hypothesize that bile acids at high concentrations stimulate adrenal cortex function: (a) cholestatic patients with significantly elevated serum bile acid levels showed significantly increased cortisol serum levels compared to control patients without cholestasis;^9^ (b) it is well known that patients with jaundice because of cholestasis undergoing surgery show wound healing defects and display increased rates of sepsis and mortality, which could be related to hypercortisolism;^10^, ^11^ (c) last but not least, Philip Hench's milestone observation that jaundice is associated with stop of disease progression in rheumatoid arthritis led finally to the discovery of cortisol for which he was awarded the Nobel Prize in 1959 together with Kendall and Reichstein.^12^

Bile acids can influence circulating glucocorticoid levels via several pathways. Interaction of bile acids with glucocorticoid metabolism takes place either at the level of catabolism in the liver or through interaction with hypothalamic CRH secretion as shown in animal models of cholestasis. Specifically, bile acids were shown to inhibit enzymes involved in cortisol breakdown such as 5‐beta reductase, 11‐beta hydroxysteroid dehydrogenase I and II in rat liver and kidney^13^, ^14^ respectively. Suppression of stress responsiveness of HPA axis^15^ as well as a defective CRH‐mediated response^16^ was observed in rat cholestatic models. In addition, bile acids can enter the brain by apical sodium‐dependent bile acid transporter (ASBT) and suppress the transcript and protein level of CRH,^17^ which may account for the suppression of the HPA axis. However, the specific role of bile acids in directly regulating steroidogenesis in the adrenal cortex remains enigmatic but appears likely under cholestatic situations, since the bile acid receptors FXR, TGR5 and S1PR2 are expressed in adrenal cortex cells.^18^, ^19^, ^20^

In this study, we consequently aimed to unravel the direct regulatory effects of bile acids on adrenal steroidogenesis and the underlying signalling pathways with the combined aid of mouse models and in vitro experiments using human adrenocortical H295R cells. Based on our experimental results, we herein provide novel direct evidence that bile acids increase steroidogenesis in mouse adrenals. In addition, we show that bile acids boost cortisol synthesis in H295R cells via an S1PR2‐ERK‐SF‐1‐dependent pathway. Therefore, our combined experimental results indicate a critical role for bile acids in stimulating adrenal gland cortex and may have important implications for patients with cholestatic liver diseases.

## 2 MATERIALS AND METHODS

### 2.1 Animal studies

All animal experiments were approved by the local authorities and were in conformity with according to the criteria outlined in *Guide for the Care and Use of Laboratory Animals* prepared by the U.S. National Academy of Science (National Institutes of Health publication 86‐23, revised 1985). FXR and TGR5 wild‐type and knockout C57/BL6 mice were obtained from a colony at the National Institutes of Health (NIH, Bethesda, MD, USA)^2^ and from the Institut de Génétique et Biologie Moléculaire et Cellulaire, Illkirch, France^21^ respectively. Animals were housed in cages together with up to four animals at the Center for Medical Research in Graz with a 12 hours’ light (6:00‐18:00) and 12 hours’ dark cycle (18:00‐6:00). Common bile duct ligation (CBDL) and sham operation in 13‐ to 20‐week‐old male C57/BL6 mice were performed for 7 days or 3 weeks as described previously.^22^ In addition to standard chow diet and water to which all animals had access ad libitum, another group of mice were fed with a 1% CDCA‐supplemented diet for 5 days. All animal experiments contained 3‐6 mice per group. Mice were sacrificed in the end by cervical dislocation in the afternoon around 18:00. Trunk blood was collected by decapitation. Adrenals were carefully removed, excised from surrounding fat and stored at liquid nitrogen for further experiments. Mouse serum was examined for the concentrations of alanine transaminase (ALT) and bile acids on a Hitachi 917 analyzer. For serum corticosterone level determination, we used a mouse/rat corticosterone ELISA (Enzo Life Sciences, Lausen, Switzerland) according to the manual.

### 2.2 Cell culture

H295R cells were obtained from the American Type Culture Collection (ATCC CRL‐2128). MTT assays were used to assess cell viability. Details are provided in the supplementary Materials and Methods section. Cells were starved with serum‐free media overnight. Taurochenodeoxycholic acid (TCDCA) (Sigma‐Aldrich, Vienna, Austria) dissolved in PBS ranging from 100 to 400 μmol/L, JTE‐013 (Cayman, Ann Arbor, MI, USA), U0126 (Cayman), Rp‐isomer (Enzo Life Sciences) or AC45594 (Tocris, Abingdon, UK) was added into the media as indicated. Cells were incubated for another 24 or 48 hours before harvesting. Cortisol levels in the medium supernatant were measured with ADVIA Centaur XP Immunoassay System (Siemens, Munich, Germany).

### 2.3 Ex vivo culture of mouse adrenals

Adrenal glands were excised from euthanized male mice and incubated with TCDCA for 2 hours. Details are provided in the supplementary Materials and Methods section.

### 2.4 Human materials

Human adrenal glands were excised from deceased, brain‐dead organ donors during the explant surgery, removed from fat and stored in liquid nitrogen. The protocol was approved by the Institutional Review Board of the Medical University of Graz.

### 2.5 Quantitative real‐time PCR (RT‐PCR)

RNA extraction was performed using TRIzol^®^ (Invitrogen, Carlsbad, CA, USA) according to the manual. cDNA samples were subjected to real‐time PCR. Specific primers are listed in Table 1. Cyclophilin or 18S rRNA was used as the reference and housekeeping genes. Details are provided in the supplementary Material and Methods section.

**Table 1:** Primer list

Gene	Accession number	Primers
mouse_c*yclophilin*	NM_011149	5′‐GGAGATGGCACAGGAGGAA‐3′
		5′‐GCCCGTAGTGCTTCAGCTT‐3′
mouse_*Star*	NM_011485	5′‐CCAGGAAGGCTGGAAGAAGG‐3′
		5′‐GTCTACCACCACCTCCAAGC‐3′
mouse_*Hsd3b1*	NM_001304800	5′‐TCCACACTGCTGCTGTCATT‐3′
		5′‐AGATGAAGGCTGGCACACTT‐3′
mouse_*Cyp21a1*	NM_009995	5′‐TCCAAGAGAGTCGGGACCAT‐3′
		5′‐CTTTCCATTGGCCTGCAACC‐3′
mouse_*Cyp11b1*	NM_001033229	5′‐CTGGGACGTGGTGTGTTCTT‐3′
		5′‐CCCTTGCTATCCCATCCACC‐3′
human*_S1PR2*	NM_004230	5′‐TCTCTACGCCAAGCATTATGTGC‐3′
		5′‐TGGCCAACAGGATGATGGA‐3′
human_*STAR*	NM_000349	5′‐TTGCTTTATGGGCTCAAGAATG‐3′
		5′‐GGAGACCCTCTGAGATTCTGCTT‐3′
human_*HSD3B2*	NM_001166120	5′‐GCGGCTAATGGGTGGAATCTA‐3′
		5′‐CCTCATTTATACTGGCAGAAAGGAAT‐3′
human_*CYP21A2*	NM_001128590	5′‐TCCCAGCACTCAACCAACCT‐3′
		5′‐CAGCTCAGAATTAAGCCTCAATCC‐3′
human_*FXR*	NM_001206979	5′‐AGGGGTGTAAAGGTTTCTTCAGGA‐3′
		5′‐ACACTTTCTTCGCATGTACATATCCAT‐3′
human_*TGR5*	NM_001077191	5′‐GCTGCTTCTTCCTGAGCCTA‐3′
		5′‐GTTGGGAGCCAAGTAGACGA‐3′
human_*18S RNA*	NR_003286	5′‐CTCAACACGGGAAACCTCAC‐3′
		5′‐AGACAAATCGCTCCACCAAC‐3′

### 2.6 Western blot

Protein extracts were subjected to SDS‐PAGE and transferred to nitrocellulose membranes. Antibodies used in this study containing STAR (Santa Cruz, Dallas, TX, USA), CYP21 (Santa Cruz), CYP11B (Santa Cruz), total ERK 1/2 (Cell Signaling Technology, Danvers, MA, USA), phosphorylation of ERK1/2 at Thr202/Tyr204 (Cell Signaling Technology), S1PR2 (Santa Cruz, Dallas, TX, USA and Proteintech, Rosemont, IL, USA), FXR (R&D Systems, Minneapolis, MN, USA), GAPDH (Santa Cruz, USA) and β‐Actin (Sigma). Membranes were incubated overnight at 4°C with respective primary polyclonal antibodies followed by 1‐hour incubation with a peroxidase‐conjugated anti‐rabbit or anti‐mouse IgG. Quantification was performed using Image Lab software from Bio‐Rad.

### 2.7 siRNA transfection

ON‐TARGET plus SMART pool siRNA primers specific to S1PR2 were synthesized by Dharmacon, USA (Catalog: J‐003952). siRNA buffer and DharmaFECT transfection reagent 1 were applied. Transfection assays were performed according to the manual provided by Dharmacon siRNA transfection protocol. After incubation for 48 hours, transfection media were removed and replaced with growth media with bile acid treatments.

### 2.8 Luciferase activity assay

SF‐1/LRH‐1 luciferase reporter was a kind gift from Prof. David D Moore (Baylor College of Medicine, Houston, TX, USA). Its promoter sequence before luciferase coding region contains SF‐1‐responsive elements. H295R cells were co‐transfected with 200 ng SF‐1 luc and β‐galactosidase reporter vectors per well, followed by incubation with 200‐300 μmol/L TCDCA for another 24 hours. Luciferase and β‐galactosidase activities were determined with Luciferase Assay System and β‐galactosidase Enzyme Assay System (Promega, Madison, WI, USA) according to manuals. Luciferase activity values were normalized to β‐galactosidase activity for internal control.

### 2.9 Statistical analysis

Unpaired Student's *t* test was conducted for comparisons between two groups. Multiple comparisons were conducted by one‐way ANOVA followed by Holm‐Sidak's multiple comparisons test for post hoc analysis with GraphPad Prism 5.0. Differences were considered statistically significant with a *P *< 0.05.

## 3 RESULTS

### 3.1 Common bile duct ligation (CBDL) and chenodeoxycholic acid (CDCA) feeding induce steroidogenesis in mice independent of FXR and TGR5

**Figure 1:**
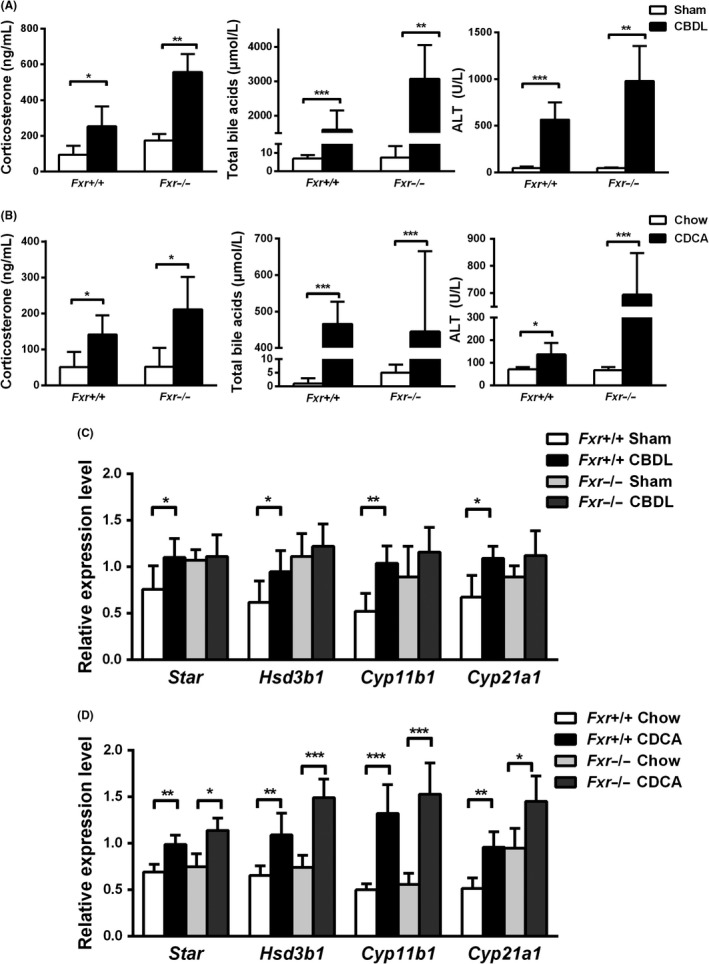
Common bile duct ligation (CBDL) and chenodeoxycholic acid (CDCA) feeding increased circulating corticosterone levels and adrenal steroidogenesis independent of farnesoid X receptor (FXR) in mice. Wild‐type (Fxr^+/+^) and Fxr knockout (Fxr^−/−^) C57BL/6 mice were subjected to *common bile duct ligation* for 7 days or fed a 1% CDCA‐supplemented diet for 5 days (n = 3‐5 per group). CBDL (A) and CDCA feeding (B) increased serum corticosterone levels independent of FXR. Elevated total serum bile acid and alanine transaminase (ALT) concentrations demonstrate the effectiveness of both treatments. mRNA levels of steroidogenesis‐related genes in mouse adrenals were normalized to cyclophilin and show an FXR‐independent increase upon CBDL (C) and CDCA feeding (D). All values are presented as means and standard deviations. Statistically significant differences are indicated by *P < 0.05, **P < 0.01, ***P < 0.001, compared to sham or chow control group

We first modelled obstructive cholestasis by CBDL in C57BL/6 wild‐type mice. Serum corticosterone levels significantly increased more than twofolds in CBDL mice compared to sham‐operated controls (Figure 1A). Since other cholephils or signalling molecules besides bile acids may directly affect adrenal steroid synthesis in CBDL, mice were fed 1% CDCA‐supplemented diet for 5 days to study the effect of a major bile acid retained in human cholestasis. Similar to CBDL, CDCA feeding also more than doubled corticosterone serum levels in mice compared to controls (Figure 1B).

**Figure 2:**
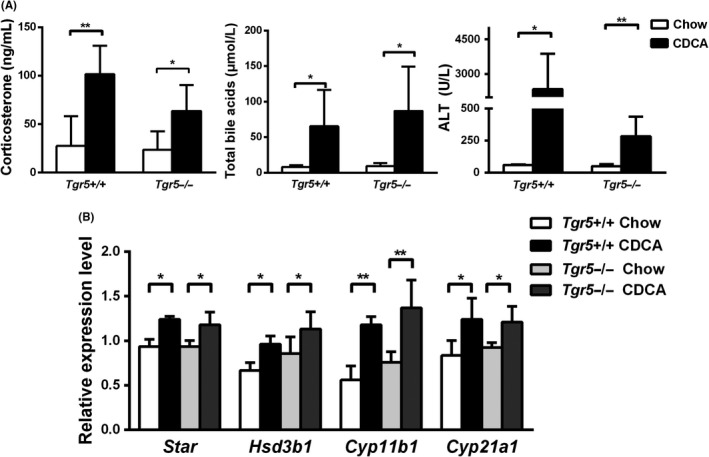
Chenodeoxycholic acid (CDCA) feeding increased circulating corticosterone levels and adrenal steroidogenesis independent of TGR5. Wild‐type (*Tgr*5^+/+^) and *Tgr5* knockout (*Tgr*5^−/−^) C57BL/6 mice were fed a 1% CDCA‐supplemented diet for 5 days (n = 3‐5 per group). CDCA feeding increased serum corticosterone levels independent of Tgr5 (A). Elevated total serum bile acid and alanine transaminase (ALT) concentrations demonstrate the effectiveness CDCA feeding. mRNA levels of steroidogenesis‐related genes in mouse adrenals were normalized to cyclophilin and show an TGR5‐independent increase upon CDCA feeding (B). All values are presented as means and standard deviations. Statistically significant differences are indicated by *P < 0.05, **P < 0.01, compared to control chow group

FXR and TGR5 knockout mice were introduced to assess the role of these two receptors. *Fxr* ^−/−^ mice showed elevated levels of corticosterone following CBDL and CDCA feeding in comparison to sham‐operated and chow‐fed mice respectively (Figure 1A,B). Corticosterone levels were even higher as in CDCA‐treated or CBDL wild‐type mice. In addition, feeding *Tgr*5^−/−^ mice with a 1% CDCA‐enriched diet also significantly increased circulating corticosterone levels compared to *Tgr5* ^−/−^ chow‐fed diet mice (Figure 2A). CBDL and CDCA feeding in mice significantly induced adrenal *Star*,* Hsd3b1*,* Cyp11b1* and *Cyp21a1* mRNA expression (Figure 1C). Notably, changes were independent of FXR and TGR5 (Figures 1C,D and 2B). Western blot analysis in addition showed elevated protein expression levels of CYP11B1 and CYP21A1 in 3‐week CBDL mice (Figure S1A,B). Taken together, these results strongly argue for the concept that bile acids induce corticosterone secretion in CBDL and CDCA‐fed mice independent of FXR and TGR5.

### 3.2 Bile acids boost cortisol secretion and regulate expression of steroidogenic enzymes in human adrenocortical H295R cells

**Figure 3:**
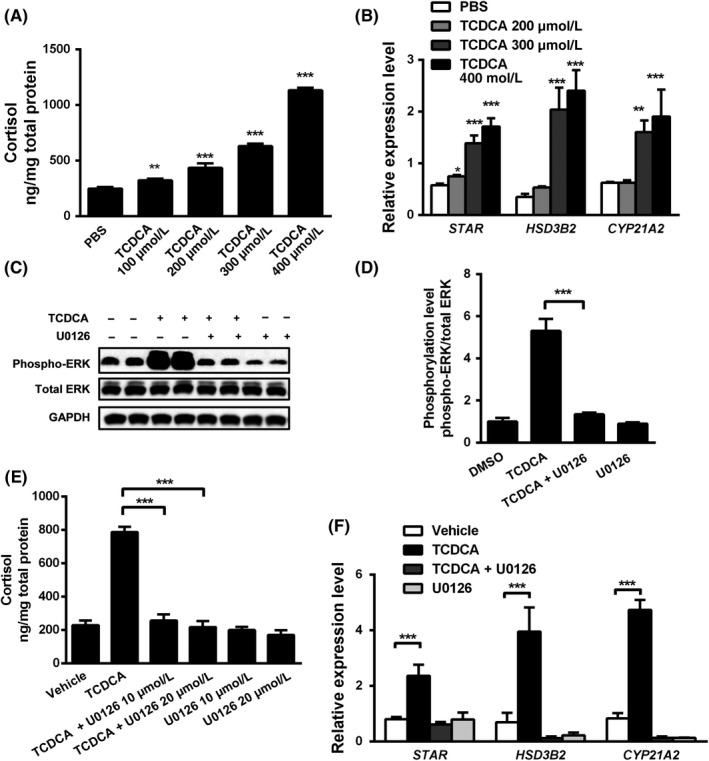
Taurochenodeoxycholic acid (TCDCA) induced steroidogenesis in H295R cells in an farnesoid X receptor (ERK)‐dependent manner. Human adrenal carcinoma cells H295R were treated for 48 hours with increasing concentrations of TCDCA. Cortisol secreted into the supernatant (normalized to total protein of cultured cells) (A) and mRNA levels of *STAR*,*CYP21A2* and *HSD3B2* (B) increased in a dose‐dependent manner in response to TCDCA challenge. H295R cells were incubated with 400 μmol/L TCDCA and 10 or 20 μmol/L U0126 (ie a specific MEK inhibitor blocking phosphorylation of ERK) for 24 hours (C, D) or 48 hours (E, F). TCDCA substantially induced phosphorylation of ERK, which was blocked by U0126 as demonstrated by Western blot analysis (C). Quantification of ERK phosphorylation compared to total ERK was performed based on four independent assays (D). Blocking ERK phosphorylation inhibited TCDCA‐induced cortisol secretion into medium (E). U0126 significantly abrogated TCDCA‐mediated induction of *STAR*,*HSD3B2* and *CYP21A2* mRNA levels (F). All mRNA levels were normalized to 18S rRNA. Values are presented as means and standard deviations. Statistically significant changes are indicated by *P < 0.05, **P < 0.01 and ***P < 0.001, compared to vehicle control group (A, B, F) or TCDCA treatment group (D, E). n = 3‐4 per group

To test direct bile acid‐dependent activation of steroidogenesis independent of other systemic factors (eg increased levels of pro‐inflammatory cytokines, changes in the whole hypothalamus‐pituitary‐adrenal gland axis), we studied the sole effects of CDCA, the most abundant bile acid in humans, in human adrenocortical H295R cells. Taurine‐conjugated CDCA (TCDCA) significantly raised cortisol synthesis in a dose‐dependent manner ranging from 100 to 400 μmol/L in this model system (Figure 3A). A concentration of 400 μmol/L was chosen for further experiments, since this high dose had the most profound effects on cortisol secretion without causing significant cell toxicity as assessed by MTT assays (Figure S2). Moreover, total serum bile acids reach levels as high as 300 μmol/L in patients with cholestasis.^5^, ^6^ In line with our in vivo findings, neither the FXR agonist INT‐747, the TGR5 agonist INT‐777 nor the combination of both induced cortisol secretion in H295R cells (Figure S3), although both receptors being expressed in H295R cells (Figure S4), supporting the concept that the herein observed effects of bile acids are independent of FXR and TGR5. Comparable to our findings in the animal models, TCDCA significantly stimulated *STAR*,* CYP21A2* (homologue of mouse *Cyp21a1*) and *HSD3B2* (homologue of mouse *Hsd3b1*) mRNA expression in H295R cells in a dose‐dependent manner (Figure 3B). Detection of increased protein levels of STAR was also consistent with the RT‐PCR results (Figure S1B,C).

Under physiological conditions, ACTH binding to its receptor activates the PKA pathway and consequently stimulates steroidogenesis in adrenal glands.^23^ However, H295R cells pretreated with the PKA inhibitor Rp‐isomer neither showed substantial reduction in cortisol secretion induced by TCDCA nor did PKA inhibition interfere with the TCDCA's effects on mRNA expression of *STAR*,* CYP21A2* and *HSD3B2* (Figure S5). These findings indicate that bile acid‐induced steroidogenesis is not mediated by PKA.

### 3.3 TCDCA triggers phosphorylation of ERK, which is pivotal for bile acid‐induced cortisol secretion

Alternatively to cAMP‐PKA, the MEK‐ERK (ie the mitogen‐activated protein kinase ‐extracellular signal‐regulated kinase) pathway is prominently linked to steroidogenesis.^24^ Consequently, we next tested the phosphorylation of ERK1/2 in response to conjugated bile acids. Treatment with 50‐400 μmol/L TCDCA enhanced ERK phosphorylation in H295R cells and 10 μmol/L MEK1/2 inhibitor U0126 co‐treatment blocked ERK phosphorylation (Figure 3C,D; Figure S6A). This finding could be reproduced in mouse adrenals cultured ex vivo and incubated with TCDCA for 2 hours (Figure S6B). When H295R cells were incubated with 10 μmol/L U0126, induction of cortisol secretion and mRNA levels of *STAR*,* HSD3B2* and *CYP21A2* by TCDCA was completely abolished (Figure 3E,F). STAR protein levels were not increased upon U0126 co‐treatment in contrast to TCDCA treatment alone (Figure S1B). Taken together, these findings demonstrate that TCDCA triggers phosphorylation of ERK in mouse adrenals ex vivo and human H295R cells in vitro, indicating a central role for ERK phosphorylation in bile acid‐induced steroidogenesis in adrenals.

### 3.4 Sphingosine‐1‐phosphate receptor 2 modulates bile acid‐induced cortisol secretion

**Figure 4:**
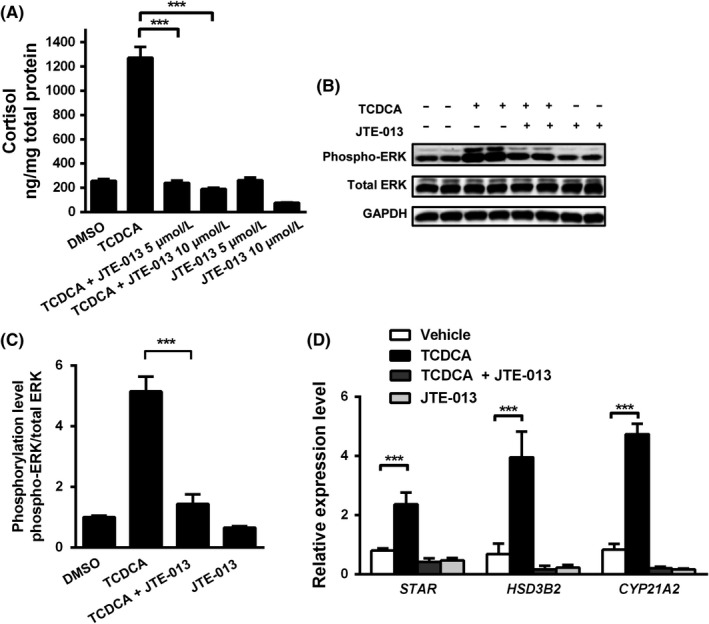
Inhibition of sphingosine‐1‐phosphate receptor 2 (S1PR2) blocked the effect of taurochenodeoxycholic acid (TCDCA) on cortisol secretion and steroidogenesis in H295R cells. H295R cells were incubated with 400 μmol/L TCDCA for 48 hours. JTE‐013 (5 and 10 μmol/L), a specific S1PR2 antagonist, was added as indicated. JTE‐013 treatment suppressed TCDCA‐induced cortisol secretion (A). (B‐D): 10 μmol/L of JTE‐013 was applied together with 400 μmol/L of TCDCA. JTE‐013 significantly reduced TCDCA‐induced ERK phosphorylation (B, C) and mRNA levels of *STAR*,*CYP21A2* and *HSD3B2* (D). mRNA levels were normalized to 18S rRNA. Induction of STAR protein levels by TCDCA was abolished by JTE‐013 treatment as detected on Western blot assays (E). Statistically significant changes are indicated by ***P < 0.001, compared to TCDCA treatment group (A, C) or vehicle control (D). Values are presented as means and standard deviations. n = 3‐4 per group

**Figure 5:**
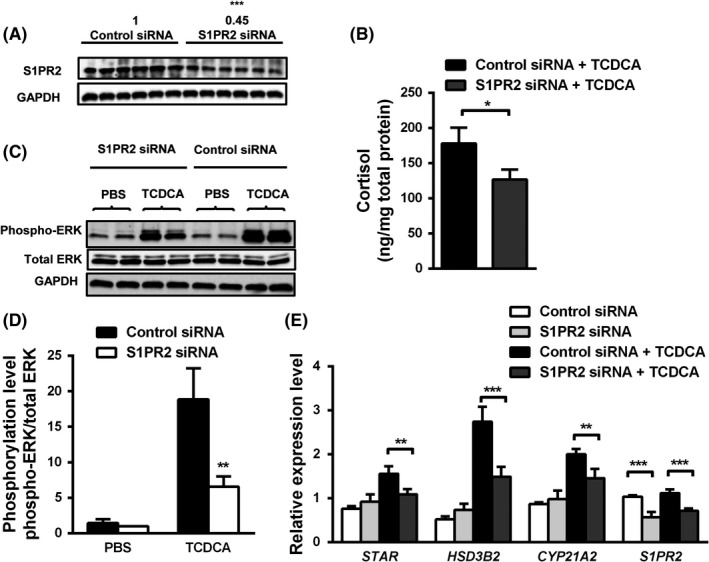
Transfection of H295R cells with sphingosine‐1‐phosphate receptor 2 (S1PR2) siRNA reduced taurochenodeoxycholic acid (TCDCA)‐induced ERK phosphorylation and steroidogenesis. H295R cells were transfected with S1PR2‐specific siRNA or negative siRNA control for 48 hours. Then, the transfection medium was removed and medium with 400 μmol/L TCDCA was added and incubated with cells for another 48 hours. Western blot assays confirmed downregulation of S1PR2 protein levels in siRNA‐treated H295R cells (A). S1PR2 siRNA treatment significantly reduced TCDCA‐induced cortisol secretion into medium (B) and phosphorylation of ERK (C, D). E: S1PR2 siRNA treatment reduces TCDCA‐induced transcription abundance of *STAR*,*HSD3B2* and *CYP21A2*. (n = 4 per group). Values are presented as means and standard deviations. Statistically significant differences are indicated by *P < 0.05, **P < 0.01, ***P < 0.001, compared to control siRNA (A, D) or control siRNA plus TCDCA treatment group (B, E). n = 3‐4 per group

Sphingosine‐1‐phosphate receptor 2 (S1PR2) has recently been identified as a novel bile acid receptor and was shown to mediate phosphorylation of ERK in mouse liver.^25^ Protein expression of S1PR2 was detected by Western blot assays in mouse adrenals, H295R cells and human adrenals (Figure S7). Co‐incubation with TCDCA and JTE‐013, a specific S1PR2 antagonist, for 48 hours significantly reduced bile acid‐dependent phosphorylation of ERK and consequently cortisol secretion in H295R cells (Figure 4A,B,C). JTE‐013 treatment also abolished TCDCA‐induced transcript abundance of *STAR*,* HSD3B2* and *CYP21A2* mRNA (Figure 4D) and STAR protein levels (Figure S1C). For additional genetic loss of function experiments, H295R cells were transfected with S1PR2 siRNA resulting in 45% knockdown (Figure 5A). In line with our chemical inhibitor experiments, TCDCA‐induced cortisol secretion, phosphorylation of ERK and mRNA levels of steroidogenesis‐related genes in siRNA‐transfected cells were also reduced significantly (Figure 5B‐E).

### 3.5 TCDCA induces steroidogenesis by elevating steroidogenic factor 1 (SF‐1) transactivation activity

**Figure 6:**
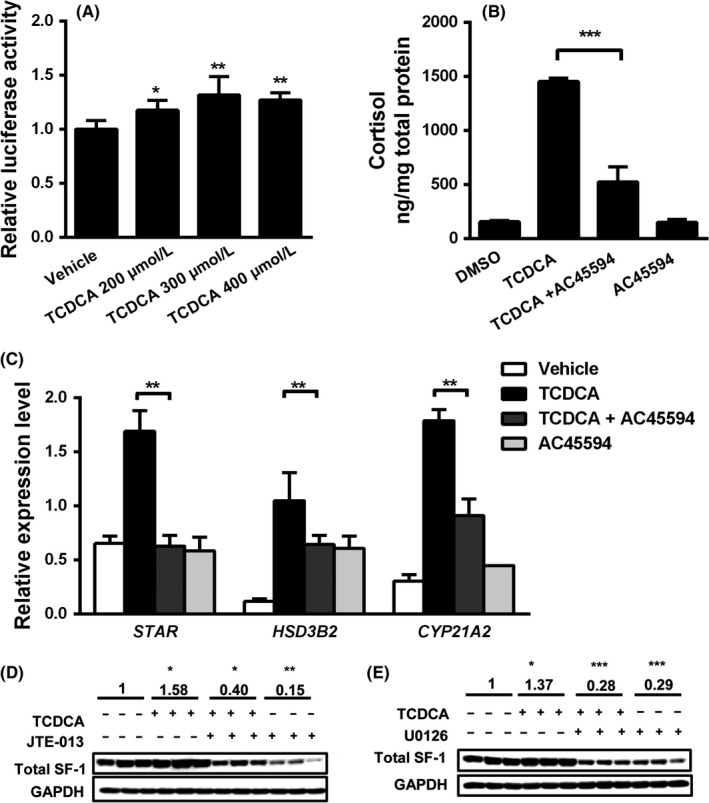
Taurochenodeoxycholic acid (TCDCA) induced steroidogenesis in H295R cells via steroidogenic factor 1 (SF‐1). H295R cells were transfected with pcDNA plasmids containing luciferase reporter genes with promoter regions responsive to SF‐1. Cells were then incubated with 200‐400 μmol/L TCDCA. Luciferase activity was normalized to β‐galactosidase activity. Each group contains three independent samples. TCDCA enhanced SF‐1 transcription activity (A). H295R cells were incubated with 400 μmol/L TCDCA and 60 μmol/L AC45594, a specific SF‐1 inverse agonist, for 48 hours. AC45594 inhibited TCDCA‐induced cortisol secretion (B) and abrogated TCDCA‐induced mRNA expression of STAR, HSD3B2 and CYP21A1 (C). Each group contains four independent samples. D, E: H295R cells incubated with 400 μmol/L TCDCA in combination with 10 μmol/L JTE‐013 or 10 μmol/L U0126 for 48 hours. SF‐1 protein levels were normalized to GAPDH. SF‐1 protein was repressed upon JTE‐013 and U0126 treatment. Statistically significant differences are indicated by *P < 0.05, **P < 0.01 and ***P < 0.001, compared to vehicle group (A, D, E) or TCDCA treatment group (B, C). n = 3‐4 per group

The transcription factor steroidogenic factor 1 (SF‐1) activity is enhanced upon phosphorylation of ERK and regulates adrenal steroidogenesis.^26^ To study whether SF‐1 participates in conjugated bile acid‐dependent induction of steroidogenesis, we performed luciferase activity assay in H295R cells using an SF‐1 luc reporter. 200‐400 μmol/L TCDCA increased SF‐1 transactivation activity in H295R cells (Figure 6A). However, when H295R cells were incubated with 400 μmol/L TCDCA plus AC45594, a SF‐1 inverse agonist, TCDCA‐induced cortisol secretion was reduced by 65% (Figure 6B). The treatment with AC45594 also successfully abolished the enhanced mRNA levels of *STAR*,* HSD3B2* and *CYP21A2* (Figure 6C). Interestingly, while 400 μmol/L TCDCA slightly elevated the basal SF‐1 protein, SF‐1 protein expression levels in H295R were substantially reduced after treatment with the ERK phosphorylation inhibitor U0126 and the S1PR2 inhibitor JTE‐013 (Figure 6D,E). These data suggest that conjugated bile acids induce steroidogenesis in H295R cells by facilitating SF‐1 transactivation activity and SF‐1 is an important link between the S1PR2‐ERK pathway and steroidogenesis.

## 4 DISCUSSION

While bile acids have been demonstrated to regulate adrenal gland function at the level of central nervous and also to affect cortisol breakdown in the liver, the question whether bile acids directly modulate adrenocortical cortisol production has so far not been addressed in detail. Bile acids are important endogenous ligands for the receptors FXR or TGR5, both being expressed in adrenals.^18^, ^19^ Therefore, we hypothesized that bile acids regulate adrenocortical steroid synthesis and secretion in an FXR‐ or TGR5‐dependent manner. Our current data, however, clearly demonstrate that CBDL and CDCA‐fed FXR as well as CDCA‐fed TGR5 knockout mice have significantly increased serum corticosterone levels thus excluding a role for these two receptors in vivo. Application of the agonists for FXR (INT‐747) and TGR5 (INT‐777) failed to increase cortisol secretion in vitro. Based on these experimental findings, we conclude that neither FXR nor TGR5 plays a pivotal role in the regulation of adrenal steroid synthesis by bile acids.

Bile acid effects may not only be mediated by signal cascades related to specific receptor activation but may also induce the inflammasome or be the simple consequence of toxicity or cell damage.^27^ We, therefore, correlated our in vivo findings with in vitro studies performed in H295R cells to investigate whether our results can experimentally be nailed down to direct effects of bile acids on the adrenal glands. Being well aware that we do not investigate the physiologic influence of bile acids on glucocorticoid production, we used supraphysiological but pathophysiologically relevant high concentrations of CDCA, one major bile acid species in humans to model a cholestatic condition in vitro. S1PR2 is activated by conjugated bile acids.^25^ The signalling of conjugated bile acids via S1PR2 has not yet been fully elucidated and is mainly based on observations in hepatocytes and cholangiocytes.^4^, ^25^, ^28^ TCDCA at supraphysiologic concentrations induced corticosteroid secretion as well as gene expression of steroidogenesis‐related genes in H295R cells in a dose‐dependent manner. These effects appear to depend on S1PR2, since S1RP2 inhibition attenuated TCDCA‐induced downstream signalling via the ERK pathway, which is in line with a previous report from rat hepatocytes using conjugated bile acids.^25^ S1PR2 activation does probably not directly lead to ERK phosphorylation but is mediated indirectly via the epidermal growth factor receptor (EGFR) as suggested in a previous study.^29^, ^30^ The overall importance of the ERK signalling pathways for our findings is highlighted by separate experiments, which show that direct inhibition of ERK abrogated TCDCA‐induced cortisol production and transcriptional effects. Consequently, this links the effects of bile acids in the adrenals to S1PR2 and ERK. The potential downstream transcription factor for ERK signalling is SF‐1, a nuclear transcription factor, which is well established to regulate the transcription of a wide range of steroidogenesis‐related enzymes including STAR, HSD3B2 and CYP21.^31^, ^32^ ERK directs the transactivation activity of SF‐1 in steroidogenic tissues.^33^ In our study, TCDCA increased SF‐1 transactivation activity, which at least to our knowledge, has not been noted before. Application of an inverse SF‐1 agonist abrogated TCDCA‐induced steroidogenic gene transcription and cortisol secretion. Protein levels of SF‐1 were substantially reduced after S1PR2 and ERK inhibition. This could be related to reduced SF‐1 protein stability because of reduced ERK phosphorylation, a mechanism which has been described for PKA‐mediated phosphorylation of SF‐1 before.^34^ Together, our data clearly demonstrate that inhibition of the S1PR2‐MEK‐ERK‐SF‐1 pathway at several signalling levels abrogates the effects of TCDCA on adrenocortical cells, highlighting the importance of this distinct pathway in bile acid‐induced steroidogenesis.

Our experimental findings may have potential clinical implications for patients with cholestatic liver diseases and jaundice. We want to emphasize that we do not provide evidence that bile acids regulate adrenal gland function under physiologic conditions. However, during cholestasis characterized by markedly elevated bile acid levels, bile acids may indeed become an important factor causing dysregulation of corticosteroid balance. One might also speculate that osteoporosis, which is frequently encountered in various cholestatic diseases, may be related to elevated serum bile acid levels as an additive explanation to reduced intestinal vitamin D absorption. Whether the changes in adrenal gland function observed in our animal models are also present in long‐standing cholestasis in humans, where multiple mechanisms along the HPA axis could counteract direct effects of bile acids on the adrenal cortex, remain to be determined.

In summary, we demonstrate for the first time that cholestasis and bile acids at pathophysiological and high concentrations can directly modulate adrenal gland function. We show increased corticosterone and cortisol production in two different cholemic mouse models and in human adrenocortical cells respectively. These effects are independent of the classical bile acid receptors FXR and TGR5 but depend on the bile acid receptor S1PR2 and downstream ERK and SF‐1.

## CONFLICT OF INTEREST

The authors declare that they have no conflicts of interest with the contents of this article.
