# Supplementary figures and images for: Bile acids increase steroidogenesis in cholemic mice and induce cortisol secretion in adrenocortical H295R cells via S1PR2, ERK and SF‐1
Source: Liver Int. 2019 Feb 17;39(11):2112–23. doi: 10.1111/liv.14052 (PMC6899711; doi:10.1111/liv.14052)

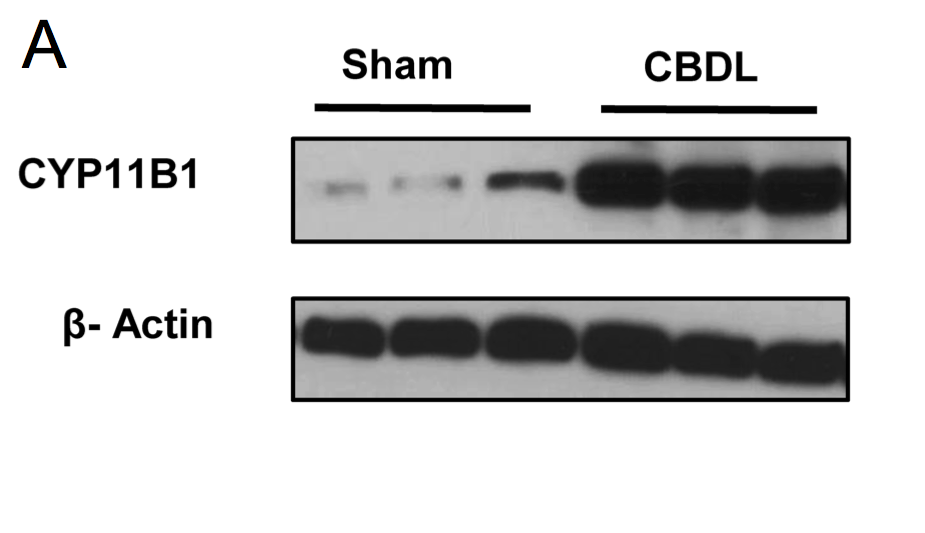

Supplement: Supplementary file 1 [file LIV-39-2112-s001.tiff]

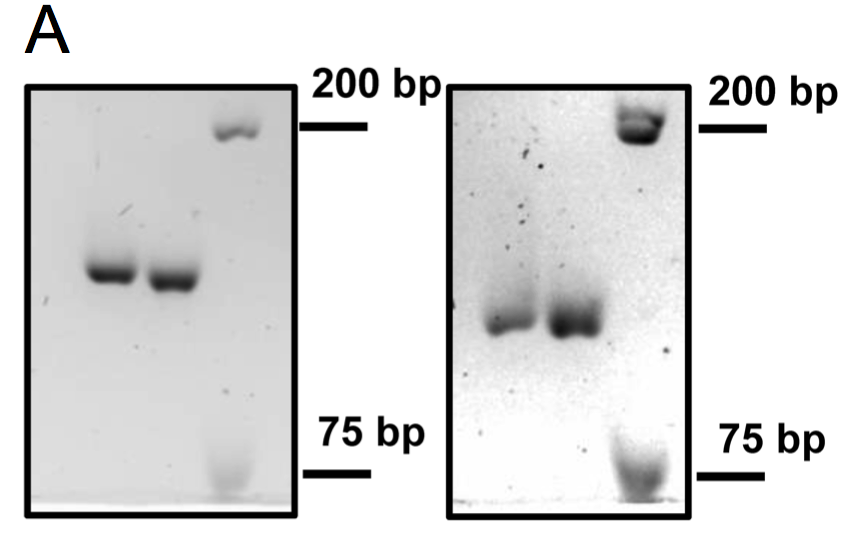

Supplement: Supplementary file 4 [file LIV-39-2112-s004.tiff]

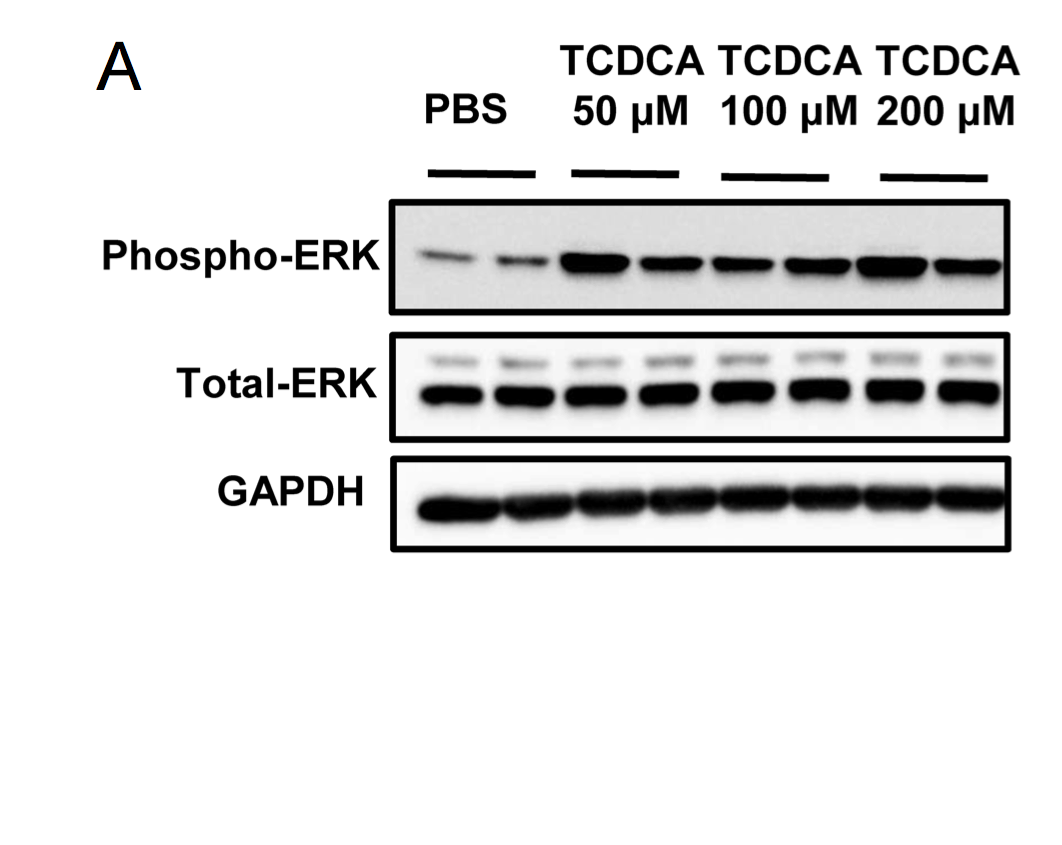

Supplement: Supplementary file 6 [file LIV-39-2112-s006.tiff]

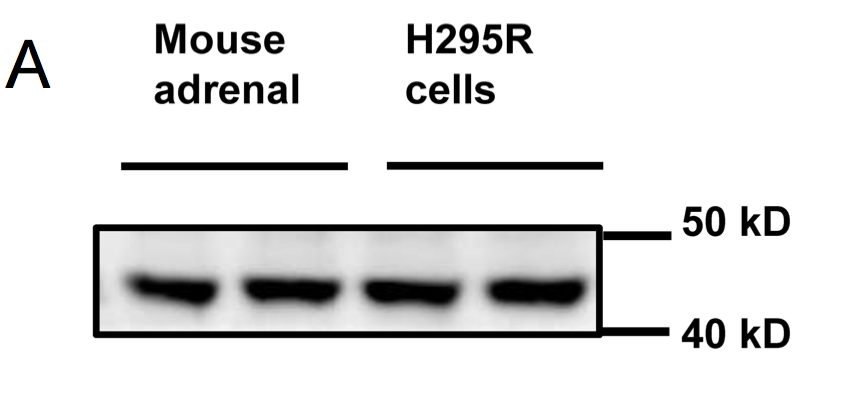

Supplement: Supplementary file 7 [file LIV-39-2112-s007.tiff]
